# How People Take Cold

**Published:** 1861-03

**Authors:** 


### HOW PEOPLE TAKE COLD.

Not by tumbling into the river and draggling home wet as a drowned rat; not by being pitched into the mud, or spilled out in the snow in sleighing time; not by walking for hours over shoe-top in mud; not by soaking in the rain without an umbrella; not by scrubbing the floor until the un-nameable sticks to you like a wet rag; not by hoeing potatoes until you are in a lather of sweat; not by trying to head a pig in mid-winter, and induce him to run the other way, for he won't do any such thing; not by steaming over the wash-tub; not by essaying to teach Biddy to make mince pies for Christmas, when you don't know how yourself, and then worrying yourself into a perspiration because the pies stuck to the pan, and came out in a muss, forgetting that pie-pans, like people, are rather the better for a little greasing, alias soft soap; these are not the things which give people colds; and yet people are all the time telling us how they "caught their death by exposure." Horace Greeley once said, "O for a leisure week to read books." Horace was green then—some say, he is now—

but I rather guess not; he is great, specially on people "of the color of black," as our three year old once described a born African. Greeley hasn't derived his greatness from books, and now he is older, perhaps he don't sigh for a week of leisure to read books, at least I don't. All the leisure I want is to think and play with the children; Bob and our new little Alice, for example. Books don't feed me, as of yore. Sure I must be getting old or hard to please; books, somehow or other, don't seem to me to meet the wants of the age, they are written too much with a view to make a sensation or money, and consequently nine out of ten fail to do either; the only result being to elucidate their authors into obscurity. Somehow or other, the mind wanders. I have to start on a journey of eight hundred miles to-morrow night and back, and the inexorable printer wants copy, and I must come back to colds; and speaking of the emptiness of books, I was wondering if "in the whole course of my life," I had ever seen defined in clear decisive phrase, in any book, "the place where and the time when" a man takes a cold. Pat, when asked one wintry day, what he would take to climb up the court-house steeple and remain there, said, "I would take a cold, yer honor." Sawney, who stood by, said he would take a dollar. That is about the nearest description I have seen in print as to the locality best adapted for taking a cold, but that was a falsity, not a fact. The seeds of a million deaths of the beautiful, the honored and the good, will be sown this year by indifference to the statement I am going to make in reference to the time and manner of taking colds. I will not now perplex the reader with a disquisition on the physiology of colds, but will simply bring to mind what any reader will recognize as an old but forgotten acquaintance.

*The* TIME *for taking cold, is after your exercise; the* PLACE *is in your own house, or office, or counting-room.*

It is not the act of exercise which gives the cold, but it is the getting cool too quick after exercising. For example, you walk very fast to get to the railroad station, or to the ferry, or to catch an omnibus, or to make time for an appointment; your mind being ahead of you, the body makes an over effort to keep up with it, and when you get to the desired spot, you raise your hat and find yourself in a perspiration; you take a seat, and feeling quite comfortable as to temperature, you begin to talk with a friend, or if a New Yorker, to read a newspaper, and before you are aware of it, you experience a sensation of chilliness, *and the thing is done;* you look around to see where the cold comes, and find a window open near you, or a door, or that you have taken a seat at the forward part of the car, and it moving against the wind, a strong draft is made through the crevices. Or may be you met a friend at a street corner, who wanted a loan, and was quite complimentary, almost loving; you did not like to be rude in the delivery of the two-lettered monosyllable, and while you were contriving to be truthful, polite, and safe, all at the same time, on comes the chilly feeling from a raw wind at the street corner, or the slosh of mud and water in which, for the first time, you noticed yourself standing.

Young ladies take their colds in grandly dark parlors, unused and unfired for a week; warm enough were they almost too warm in the gay, sun-shiny street without, and that parlor felt comfortably cool at first, but the last curl of the visited would not dangle satisfactorily, and while compelling it (young ladies now a-days making it a point of principle not to be thwarted in any thing, not even in wedding rich Tom to please the old folks, when they love poor Dick, and intend to please themselves), while conquering that beautiful but unruly curl, the visiter makes an unexpected meeting with a chill which calls her to the ——— grave.

I cannot give further space to illustrations to arrest the attention of the careless, but will reiterate the principle for the thoughtful and observant:

GET COOL SLOWLY.

After any kind of exercise, do not stand a moment at a street corner, for any body or any thing; nor at an open door or window. When you have been exercising in any way whatever, winter or summer, go home at once, or to some sheltered place; and however warm the room may seem to be, do not at once pull off your hat and cloak, but wait awhile, [illegible] five minutes or more, and lay aside one at a time; thus acting, a cold is impossible. Notice a moment: when you return from a brisk walk and enter a warm room, raise your hat, and the forehead will be moist; let the hat remain a few moments and feel the forehead again, and it will be dry, showing that the room is actually cooler than your body, and that with your out-door clothing on, you have cooled off full soon. Among the severest colds I have known men to take, were the result of sitting down to a meal in a cool room, after a walk; or being engaged in writing, have let the fire go out, and their first admonition of it was that creeping chilliness which is the ordinary forerunner of a severe cold. Persons have often lost their lives by writing or reading in a room where there was no fire, although the weather outside was rather uncomfortable. Sleeping in rooms long unused, has destroyed the life of many a visitor and friend. Our splendid parlors, and our nice "spare rooms," help to enrich many a doctor. The cold sepulchral parlors of New York, from May until November, bring disease, not only to visitors, but to the visited; for coming in from domestic occupations, or from the hurry of dressing, the heat of the body is higher than natural, and having no cloak or hat on in going in to meet a visitor, and having in addition but little vitality, in consequence of the very sedentary nature of town life, there is but very little capability of resistance, and a chill and cold is the result.

But *how to cure a cold promptly?* that is a question of life and death to multitudes. There are two methods of universal application: 1st, obtain a bottle of cough mixture, or a lot of cough candy, any kind will do; in a day or two you will *feel* better, and in high spirits; you will be charmed with the promptness of the medicine; make a mule of yourself, by giving your certificate of the valuable remedy, and in due course of time, another certificate will be made for your admission, foot foremost, into "Greenwood."

The other remedy is, consult a respectable resident physician.

